# The Woman Worker's World

**Published:** 1920-07-31

**Authors:** 


### THE WOMAN WORKER'S WORLD.

### By a SOCIAL WORKER.

PROGRESS IN PROFESSIONAL WORK.

THE Federation of University Women enterprisingly speak of their "first annual" meeting at Bedford College this month. It is to be hoped that the phrase will be amply justified, for the objects they have in view are some which anyone interested in women's progress in professional work must wish to see followed.

Practically these objects are one—a deepening of the intellectual development which makes an educated and competent human being. The Conference opened with an evening reception at Bedford College, during which addresses were delivered by Lord Grey of Falloden, who is much interested, and who helped to promote the visit of a deputation of inquiry, from this country to America. Speeches were also made by Dean Virginia Gildersleeve, of Boston, by Professor Caroline Spurgeon, this year's President, and Dr. Winifred Cullis, London School of Medicine.

A WOMEN'S CLUB IN EACH COUNTRY.

For a practical beginning it is proposed that each Federation shall start a Club (in Paris Mrs. Whitelaw Reid has already given her house for this purpose) and form a Hospitality Committee. Here is an interesting point for the medical profession. Many women doctors are University graduates; but few nurses are in this enviable position. Suppose a College of Nursing of University rank; suppose, again, similar steps taken by Associations of Nurses in different countries, how pleasant might a trip abroad be, and how profitable!

Further, through the Federation of University Women interchange of students and lecturers will be facilitated. To be able to pass on for a final year in the culture of another nationality would often be a priceless boon. Hitherto English women have appreciated more than English men the value of knowing one European language at least to the point of speaking and reading easily. Some such knowledge is certainly essential to real acquaintance. But we can hardly say that a majority of fairly-well educated English girls are thus competent, even in French.

EDUCATION IN MANY LANDS.

Very interesting details were given by foreign delegates as to the position and progress of women's education in their countries. The charming Miss Nováková, from Prague University, who represented Czecho-Slovakia, related that on its formation the Republic had given women the same right of entry to studies as men have, theology being the only exception. But in this subject a daughter of President Masaryk has asked leave to lecture. Many students work now without coal and with very little oil for their rooms. Visits from people of friendly nations are greatly desired.

From Italy Signorina Cimino King told of a wonderful awakening to the wish for education among the most backward women of the country, those of the South reaching even to the peasant women of Calabrian villages. Salaries have been so grievously deficient that in 1911 a number of schools round Venice and in mountain districts were closed, the teachers preferring to become servants and waiters.

In Belgium Mlle. Vanderstichele said that education tended to be too much through books, children doing more "lessons". than in any other country. But even when carried through the University, where women are few in comparison to men, it left a need for further practical training. A pharmacist might have, for instance, to give an extra year. Women doctors, she said, have not as yet fully won public confidence.

Women pharmacist students in France are four times as many as before the war. In 1917 a decree opened the Institute of Chemistry as well as the Schools of Mining and of Electricity, and these developments are the direct reward of women's war work. In 1914 all University *Licenciées* were mobilised for State service, many being put on to replace men in research work as also in other scientific careers, and they were felt to have deserved the breaking down of barriers.

In Spain the idea of "marriage or convent" dies hard among upper-class women. But Madrid University has among its eighty-five students a duke's daughter. The country has but one woman University professor—the novelist Señorita de Bazan—though under a thirteenth century law of Alfonso the Wise all study was open to them. Convention lost the privileges, but now among 218 women at the eleven Universities 37 are studying pharmacy and 63 medicine.

PLEA FOR HOME LIFE FOR THE CHILD.

The State Children Association's Annual Meeting, held by kind permission at Lord Islington's house in Portman Square, was enlivened by a charming paper from his young daughter, the Hon. Joan Poynder, at once pathetic and amusing, and full of hope as a token of what the young people who will care for forlorn childhood can accomplish. Miss Poynder pleaded for the memory of a happy and natural childhood, spent in a home circle as the foundation, for health of mind and body in every citizen. Boarding-out needs care and wisdom, of course, but it is actually more economical as well as more humane than any herding of children in Institutions. Stress was laid by Miss Poynder on the value of the school nurse's attention to the boarded-out children in discovering tendencies to ill-health and remedies for defects. Lady Astor spoke of the ready interest taken by some of the younger members of Parliament in legislation for children, and such matters as widows' pensions; and Miss M. Fry, for the Penal Reform League, urged that in the case of a delinquent child more attention should be given to the questions of its physical and mental condition, either of which might be the true source of criminal tendencies. A physical and a mental clinic needed to be in operation.

WOMEN AND PUBLIC HEALTH.

The Public Health Committee of the National Council of Women propose a resolution for the Conference of the Council at Bristol in October, to the effect that instruction in the symptoms of venereal disease and its treatment should be given to all hospital nurses during training. They also wish to see pressure brought to bear against housing regulations which will permit the further reduction of size of bedrooms. It was stated that some rooms lately built are so small that the bed cannot be properly made if the door has to be opened while it is in use. The explanation of this rather surprising statement is probably that the poor are still buying cheaply and at second-hand the large double beds which the well-to-do have discarded as insanitary, an abuse which is likely to persist for a long time unless by-laws can deal with it.

NEED FOR STANDARDISATION IN MEDICAL INSPECTION.

At the same Conference the Emigration Committee of the Council will bring forward a resolution for the provision of a standardisation of health in medical inspection of emigrants. Some very hard cases result now from persons passed at the port of departure being rejected at the port of arrival. This resolution will also be presented to the meeting of the International Council of Women at Christiania in September.

